# Monosuccinate ester of melampomagnolide B

**DOI:** 10.1107/S1600536814002815

**Published:** 2014-02-28

**Authors:** Venumadhav Janganati, Narsimha Reddy Penthala, Nikhil Reddy Madadi, Sean Parkin, Peter A. Crooks

**Affiliations:** a Department of Pharmaceutical Sciences, College of Pharmacy, University of Arkansas for Medical Sciences, Little Rock, AR 72205, USA; b Department of Chemistry, University of Kentucky, Lexington, KY 40506, USA

## Abstract

The title monosuccinate derivative of melampomagnolide B [systematic name: 4-(((1a*R*,7a*S*,10a*S*,10b*S*,*E*)-1a-methyl-8-meth­yl­ene-9-oxo-1a,2,3,6,7,7a,8,9,10a,10b-deca­hydro­oxireno[2′,3′:9,10]cyclo­deca­[1,2-*b*]furan-5-yl)meth­oxy)-4-oxo­butan­oic acid], C_19_H_24_O_7_, was obtained from the reaction of melampomagnolide B with succinic anhydride under nucleophilic addition reaction conditions. The mol­ecule is built up from fused ten-, five- (lactone) and three-membered (epoxide) rings. The inter­nal double bond in the ten-membered ring has the *cis* geometry (*i.e.* it is the *E* isomer). The lactone ring has an envelope-type conformation, with the (chiral) C atom opposite the lactone O atoms as the flap atom. In the crystal, O—H⋯O hydrogen bonds link the mol­ecules into chains parallel to the *b-*axis direction.

## Related literature   

For the biological activity of similar compounds, see: Nasim *et al.* (2011 ▶). For the isolation of a similar compound, see: El-Feraly (1984 ▶). For the structures and syntheses of similar compounds, see: Gonzalez *et al.* (1988 ▶); Macias *et al.* (1992 ▶); Casimir *et al.* (1995 ▶). Refinement progress was checked using routines in PLATON (Spek, 2009 ▶) and by the R-tensor (Parkin, 2000 ▶) The crystal was placed directly into the cold stream of a liquid nitro­gen based cryostat, according to published methods, see: Hope (1994 ▶); Parkin & Hope (1998 ▶).
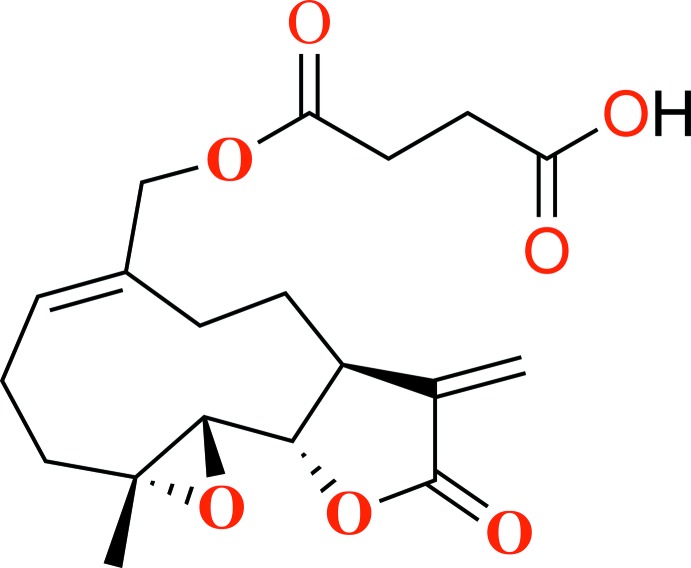


## Experimental   

### 

#### Crystal data  

C_19_H_24_O_7_
*M* *_r_* = 364.38
Orthorhombic, 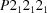

*a* = 8.7866 (2) Å
*b* = 9.6082 (2) Å
*c* = 21.0088 (5) Å
*V* = 1773.63 (7) Å^3^
*Z* = 4
Cu *K*α radiation
μ = 0.87 mm^−1^
*T* = 90 K
0.21 × 0.20 × 0.18 mm

#### Data collection  

Bruker X8 Proteum diffractometer
Absorption correction: multi-scan (*SADABS*; Sheldrick, 2008*a* ▶) *T*_min_ = 0.818, *T*_max_ = 0.889
21766 measured reflections
3218 independent reflections
3204 reflections with *I* > 2σ(*I*)
*R*_int_ = 0.033

#### Refinement  

*R*[*F* ^2^ > 2σ(*F* ^2^)] = 0.024
*wR*(*F* ^2^) = 0.062
*S* = 1.03
3218 reflections
240 parameters
H atoms treated by a mixture of independent and constrained refinement
Δρ_max_ = 0.16 e Å^−3^
Δρ_min_ = −0.15 e Å^−3^
Absolute structure: Flack parameter determined using 1346 quotients [(*I* ^+^)−(*I* ^−^)]/[(*I* ^+^)+(*I* ^−^)] (Parsons *et al.*, 2013 ▶)
Absolute structure parameter: −0.02 (3)

### 

Data collection: *APEX2* (Bruker, 2006 ▶); cell refinement: *SAINT* (Bruker, 2006 ▶); data reduction: *SAINT*; program(s) used to solve structure: *SHELXS97* (Sheldrick, 2008*b* ▶); program(s) used to refine structure: *SHELXL2013* (Sheldrick, 2008*b* ▶); molecular graphics: *XP* in *SHELXTL* (Sheldrick, 2008*b* ▶); software used to prepare material for publication: *SHELXL97* (Sheldrick, 2008*b* ▶) and *CIFFIX* (Parkin, 2013 ▶).

## Supplementary Material

Crystal structure: contains datablock(s) global, I. DOI: 10.1107/S1600536814002815/sj5379sup1.cif

Structure factors: contains datablock(s) I. DOI: 10.1107/S1600536814002815/sj5379Isup2.hkl

Supporting information file. DOI: 10.1107/S1600536814002815/sj5379Isup3.cml

CCDC reference:

Additional supporting information:  crystallographic information; 3D view; checkCIF report

## Figures and Tables

**Table 1:** Hydrogen-bond geometry (Å, °)

*D*—H⋯*A*	*D*—H	H⋯*A*	*D*⋯*A*	*D*—H⋯*A*
O7—H7⋯O1^i^	0.88 (2)	1.91 (3)	2.7616 (16)	162 (2)
C6—H6*A*⋯O6^ii^	1.00	2.60	3.173 (2)	117
C7—H7*A*⋯O1^iii^	1.00	2.40	3.3293 (19)	154
C14—H14*A*⋯O7^iv^	0.99	2.51	3.418 (2)	153

Symmetry codes: (i) 
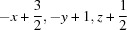
; (ii) 
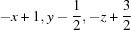
; (iii) 
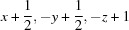
; (iv) 
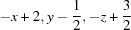
.

**Fig. 1.**
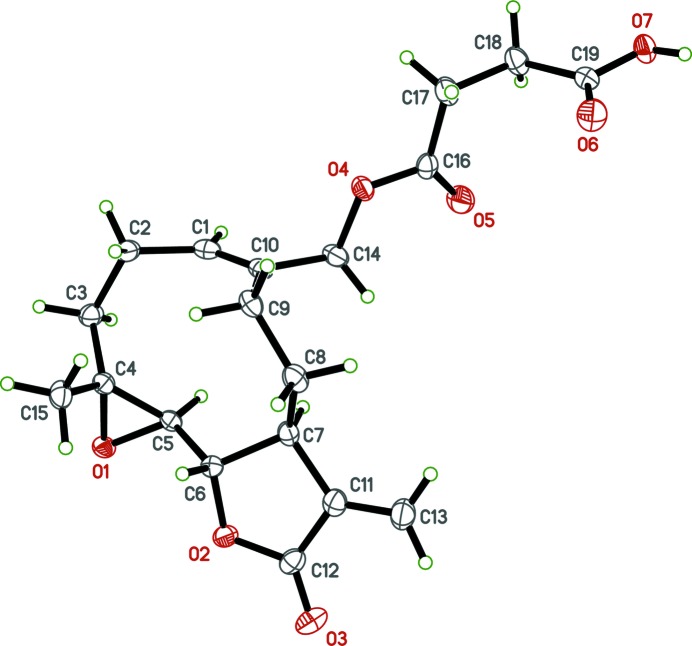
The title molecule with the atom numbering scheme. Displacement ellipsoids are drawn at the 50% probability level.

## supplementary crystallographic information

### 1. Comment

Melampomagnolide B (MMB), a melampolide originally isolated from *Magnolia grandiflora*, (El-Feraly, 1984), has been identified as a new antileukemic sesquiterpene with properties similar to parthenolide (PTL). MMB was synthesized by a method using selenium oxide (Macias *et al.*, 1992) for the oxidation of the C10 methylgroup of PTL, which also results in concomitant conversion of the geometry of the C9—C10 double bond from *E* to *Z* (Gonzalez *et al.* 1988). Recently, Nasim *et al.* (2011) have reported the biotin-conjugate derivative of melampomagnolide B, to elucidate its anti-leukemic mechanism of action. More importantly, from a drug design point of view, MMB is a more interesting molecule because it contains an OH group, which provides the means for designing pro-drugs with improved water solubility, bioavailability and tissue targeting. In the current study we synthesized a mono succinate derivative of MMB by the reaction of MMB with succinic anhydride (Casimir *et al.* 1995). The compound obtained was recrystallized from a mixture of 9:1 dichloromethane and methanol. In order to obtain detailed information on the structural conformation of this molecule a single-crystal X-ray structure determination has been carried out. This revealed that in the crystal structure the molecules of the title compound are connected by intermolecular O—H···O hydrogen bonds between the carboxylic acid (donor) and epoxide (acceptor) groups, linking the molecules into chains that propagate parallel to the *c*-axis.

### 2. Experimental

To a reaction mixture of MMB (200 mg, 0.76 mmol) and triethylamine (76.7 mg, 0.76 mmol) in dichloromethane (5 mL), succinic anhydride (76 mg, 0.76 mmol) was added at ambient temperature. The resulting reaction mixture was stirred for 48 h and the reaction was monitored by TLC. After completion of the reaction the resulting mixture was concentrated under reduced pressure to afford the crude product, which was purified by column chromatography (silica gel, 3-5% methanol in dichloromethane) to obtain the desired compound 4-(((1a*R*,7a*S*,10a*S*,10b*S*,*E*)-1a-methyl-8-methylene-9-oxo-1a,2,3,6,7,7a,8,9,10a,10b-decahydrooxireno[2',3':9,10]cyclodeca[1,2-*b*]furan-5-yl)methoxy)-4-oxobutanoic acid as a white solid (yield: 90 %). The obtained solid was recrystallized from a mixture of dichloromethane and methanol (9:1) as colourless needles. Melting point 398-399°K. ^1^H NMR (400 MHz, DMSO-d_6_): δ 12.25 (s, 1H), 6.05 (d, *J*=2.8 Hz, 1H), 5.64-5.57 (m, 2H), 4.64 (d, *J*=12.4 Hz, 1H), 4.42 (d, *J*=12.8 Hz, 1H), 4.12 (t, *J*= 9.6 Hz, 1H), 2.99 (t, *J*=3 Hz, 1H), 2.85 (d, *J*=9.6 Hz, 1H), 2.30-2.04 (m, 10H), 1.66 (t, *J*=11.6 Hz, 1H), 1.47 (s, 3H), 0.96 (t, *J*=11.6 Hz, 1H); ^13^C NMR (100 MHz DMSO-d_6_): δ 173.8, 172.4,169.8, 140.0, 135.3, 129.5, 119.7, 110.0, 81.0, 66.9, 63.0, 60.3, 42.2, 36.7, 29.1, 25.0, 24.2, 23.6, 17.9 *ppm*.

### 3. Refinement

H atoms were found in difference Fourier maps and subsequently placed at idealized positions with constrained distances of 0.98 Å (RCH_3_), 0.99 Å (R_2_CH_2_), 1.00 Å (R_3_CH), 0.95 Å (C_sp2_H). The OH hydrogen coordinates attached to O7 were freed in the final cycles of refinement. U_iso_(H) values were set to either 1.2U_eq_ or 1.5U_eq_ (RCH_3_, OH) of the attached atom.

### Crystal data

**Table:**

C_19_H_24_O_7_	*D*_x_ = 1.365 Mg m^−^^3^
*M**_r_* = 364.38	Cu *K*α radiation, λ = 1.54178 Å
Orthorhombic, *P*2_1_2_1_2_1_	Cell parameters from 9822 reflections
*a* = 8.7866 (2) Å	θ = 5.5–68.3°
*b* = 9.6082 (2) Å	µ = 0.87 mm^−^^1^
*c* = 21.0088 (5) Å	*T* = 90 K
*V* = 1773.63 (7) Å^3^	Wedge, colourless
*Z* = 4	0.21 × 0.20 × 0.18 mm
*F*(000) = 776	

### Data collection

**Table:**

Bruker X8 Proteum diffractometer	3218 independent reflections
Radiation source: fine-focus rotating anode	3204 reflections with *I* > 2σ(*I*)
Detector resolution: 5.6 pixels mm^-1^	*R*_int_ = 0.033
φ and ω scans	θ_max_ = 68.2°, θ_min_ = 5.5°
Absorption correction: multi-scan (*SADABS*; Sheldrick, 2008*a*)	*h* = −10→4
*T*_min_ = 0.818, *T*_max_ = 0.889	*k* = −11→11
21766 measured reflections	*l* = −24→25

### Refinement

**Table:**

Refinement on *F*^2^	Hydrogen site location: mixed
Least-squares matrix: full	H atoms treated by a mixture of independent and constrained refinement
*R*[*F*^2^ > 2σ(*F*^2^)] = 0.024	*w* = 1/[σ^2^(*F*_o_^2^) + (0.0321*P*)^2^ + 0.4142*P*] where *P* = (*F*_o_^2^ + 2*F*_c_^2^)/3
*wR*(*F*^2^) = 0.062	(Δ/σ)_max_ < 0.001
*S* = 1.03	Δρ_max_ = 0.16 e Å^−^^3^
3218 reflections	Δρ_min_ = −0.15 e Å^−^^3^
240 parameters	Extinction correction: *SHELXL2013* (Sheldrick, 2008a), Fc^*^=kFc[1+0.001xFc^2^λ^3^/sin(2θ)]^-1/4^
0 restraints	Extinction coefficient: 0.0033 (6)
Primary atom site location: structure-invariant direct methods	Absolute structure: Flack parameter determined using 1346 quotients [(*I*^+^)-(*I*^-^)]/[(*I*^+^)+(*I*^-^)] (Parsons *et al.*, 2013)
Secondary atom site location: difference Fourier map	Absolute structure parameter: −0.02 (3)

### Special details

**Table:**

Experimental. The crystal was mounted with polyisobutene oil on the tip of a fine glass fibre, fastened in a copper mounting pin with electrical solder. It was placed directly into the cold stream of a liquid nitrogen based cryostat, according to published methods (Hope, 1994; Parkin & Hope, 1998). Diffraction data were collected with the crystal at 90K, which is standard practice in this laboratory for the majority of flash-cooled crystals.
Geometry. All esds (except the esd in the dihedral angle between two l.s. planes) are estimated using the full covariance matrix. The cell esds are taken into account individually in the estimation of esds in distances, angles and torsion angles; correlations between esds in cell parameters are only used when they are defined by crystal symmetry. An approximate (isotropic) treatment of cell esds is used for estimating esds involving l.s. planes.
Refinement. Refinement progress was checked using routines in Platon (Spek, 2009), by the R-tensor (Parkin, 2000), and with the IUCr utility checkCIF.

### Fractional atomic coordinates and isotropic or equivalent isotropic displacement parameters (Å^2^)

**Table:**

	*x*	*y*	*z*	*U*_iso_*/*U*_eq_	
O1	0.20411 (13)	0.31754 (11)	0.44993 (5)	0.0191 (3)	
O2	0.23859 (13)	0.11877 (11)	0.55661 (5)	0.0204 (3)	
O3	0.26866 (16)	−0.07622 (12)	0.61342 (6)	0.0307 (3)	
O4	0.73762 (12)	0.64045 (12)	0.64763 (5)	0.0201 (3)	
O5	0.95459 (14)	0.52306 (14)	0.66681 (7)	0.0309 (3)	
O6	0.98811 (14)	0.66939 (14)	0.83142 (6)	0.0290 (3)	
O7	1.23791 (13)	0.70884 (13)	0.82130 (6)	0.0229 (3)	
H7	1.238 (3)	0.690 (2)	0.8623 (12)	0.034*	
C1	0.5239 (2)	0.60864 (17)	0.52307 (8)	0.0214 (4)	
H1A	0.6171	0.6045	0.5002	0.026*	
C2	0.3899 (2)	0.66538 (17)	0.48663 (8)	0.0248 (4)	
H2A	0.4240	0.7463	0.4612	0.030*	
H2B	0.3127	0.6990	0.5173	0.030*	
C3	0.3147 (2)	0.55854 (17)	0.44177 (8)	0.0223 (3)	
H3A	0.2483	0.6076	0.4110	0.027*	
H3B	0.3944	0.5087	0.4175	0.027*	
C4	0.22202 (19)	0.45571 (16)	0.47891 (7)	0.0184 (3)	
C5	0.30221 (18)	0.33451 (16)	0.50515 (7)	0.0162 (3)	
H5A	0.4138	0.3313	0.4958	0.019*	
C6	0.25592 (17)	0.26798 (16)	0.56681 (7)	0.0167 (3)	
H6A	0.1580	0.3090	0.5822	0.020*	
C7	0.38020 (17)	0.28350 (17)	0.61809 (7)	0.0184 (3)	
H7A	0.4809	0.2858	0.5960	0.022*	
C8	0.3701 (2)	0.41277 (18)	0.66040 (8)	0.0227 (4)	
H8A	0.4472	0.4042	0.6945	0.027*	
H8B	0.2688	0.4134	0.6811	0.027*	
C9	0.39325 (18)	0.55441 (17)	0.62692 (7)	0.0199 (3)	
H9A	0.3006	0.5757	0.6019	0.024*	
H9B	0.4034	0.6273	0.6600	0.024*	
C10	0.52939 (18)	0.56347 (16)	0.58308 (8)	0.0190 (3)	
C11	0.36737 (18)	0.14530 (18)	0.65197 (8)	0.0217 (4)	
C12	0.28777 (19)	0.04748 (17)	0.60846 (8)	0.0220 (4)	
C13	0.4154 (2)	0.1067 (2)	0.70887 (9)	0.0300 (4)	
H13A	0.3994	0.0140	0.7231	0.036*	
H13B	0.4660	0.1717	0.7356	0.036*	
C14	0.68183 (18)	0.52273 (17)	0.60986 (8)	0.0223 (4)	
H14A	0.6716	0.4390	0.6370	0.027*	
H14B	0.7539	0.5015	0.5750	0.027*	
C15	0.07576 (19)	0.50907 (18)	0.50694 (8)	0.0231 (4)	
H15A	0.0156	0.4307	0.5230	0.035*	
H15B	0.0178	0.5584	0.4741	0.035*	
H15C	0.0986	0.5729	0.5420	0.035*	
C16	0.87876 (18)	0.62669 (18)	0.67217 (7)	0.0197 (3)	
C17	0.9271 (2)	0.75939 (19)	0.70422 (8)	0.0267 (4)	
H17A	0.8544	0.7803	0.7389	0.032*	
H17B	0.9210	0.8362	0.6729	0.032*	
C18	1.0873 (2)	0.7560 (2)	0.73186 (8)	0.0249 (4)	
H18A	1.1513	0.6947	0.7051	0.030*	
H18B	1.1309	0.8509	0.7297	0.030*	
C19	1.09495 (19)	0.70589 (17)	0.79952 (8)	0.0189 (3)	

### Atomic displacement parameters (Å^2^)

**Table:**

	*U*^11^	*U*^22^	*U*^33^	*U*^12^	*U*^13^	*U*^23^
O1	0.0226 (6)	0.0180 (5)	0.0165 (5)	−0.0031 (4)	−0.0027 (4)	−0.0021 (4)
O2	0.0256 (6)	0.0170 (5)	0.0188 (5)	−0.0029 (5)	−0.0005 (5)	0.0013 (4)
O3	0.0416 (7)	0.0206 (6)	0.0299 (6)	−0.0002 (5)	0.0045 (6)	0.0061 (5)
O4	0.0175 (6)	0.0213 (6)	0.0215 (5)	0.0006 (4)	−0.0039 (4)	−0.0023 (4)
O5	0.0225 (6)	0.0267 (7)	0.0436 (7)	0.0041 (5)	−0.0058 (6)	−0.0024 (6)
O6	0.0205 (6)	0.0381 (8)	0.0284 (6)	−0.0032 (5)	0.0045 (5)	0.0074 (6)
O7	0.0177 (6)	0.0311 (6)	0.0200 (6)	−0.0015 (5)	−0.0027 (4)	0.0018 (5)
C1	0.0225 (8)	0.0161 (7)	0.0256 (9)	−0.0040 (7)	0.0014 (7)	−0.0039 (6)
C2	0.0335 (9)	0.0170 (8)	0.0239 (8)	−0.0047 (7)	−0.0040 (7)	0.0013 (7)
C3	0.0296 (9)	0.0193 (8)	0.0182 (7)	−0.0033 (7)	−0.0028 (7)	0.0012 (6)
C4	0.0214 (8)	0.0178 (8)	0.0160 (7)	−0.0010 (7)	−0.0036 (6)	−0.0025 (6)
C5	0.0160 (7)	0.0170 (7)	0.0156 (7)	−0.0016 (6)	−0.0004 (6)	−0.0025 (6)
C6	0.0166 (7)	0.0164 (7)	0.0171 (7)	−0.0003 (6)	0.0005 (6)	−0.0002 (6)
C7	0.0151 (7)	0.0231 (8)	0.0170 (7)	−0.0003 (6)	−0.0010 (6)	0.0014 (6)
C8	0.0230 (8)	0.0296 (9)	0.0155 (7)	−0.0026 (7)	−0.0008 (6)	−0.0023 (6)
C9	0.0172 (7)	0.0240 (8)	0.0185 (7)	0.0007 (6)	−0.0021 (6)	−0.0051 (6)
C10	0.0166 (7)	0.0156 (7)	0.0248 (8)	−0.0011 (6)	−0.0012 (6)	−0.0050 (6)
C11	0.0155 (7)	0.0267 (9)	0.0227 (8)	0.0031 (6)	0.0026 (6)	0.0035 (7)
C12	0.0218 (8)	0.0225 (9)	0.0218 (8)	0.0022 (7)	0.0052 (7)	0.0042 (6)
C13	0.0246 (9)	0.0382 (10)	0.0272 (9)	−0.0007 (8)	−0.0031 (7)	0.0105 (8)
C14	0.0175 (8)	0.0197 (8)	0.0297 (8)	−0.0019 (7)	−0.0022 (6)	−0.0050 (7)
C15	0.0218 (8)	0.0232 (8)	0.0245 (8)	0.0049 (7)	−0.0040 (7)	−0.0010 (7)
C16	0.0170 (7)	0.0254 (9)	0.0166 (7)	−0.0004 (7)	0.0011 (6)	0.0049 (6)
C17	0.0277 (9)	0.0285 (9)	0.0238 (8)	0.0007 (8)	−0.0080 (7)	−0.0025 (7)
C18	0.0230 (8)	0.0338 (9)	0.0181 (8)	−0.0042 (7)	−0.0020 (6)	−0.0002 (7)
C19	0.0171 (7)	0.0191 (7)	0.0204 (8)	−0.0013 (6)	0.0001 (6)	−0.0024 (6)

### Geometric parameters (Å, º)

**Table:**

O1—C5	1.4545 (18)	C7—C11	1.511 (2)
O1—C4	1.4691 (18)	C7—C8	1.530 (2)
O2—C12	1.357 (2)	C7—H7A	1.0000
O2—C6	1.4575 (19)	C8—C9	1.545 (2)
O3—C12	1.205 (2)	C8—H8A	0.9900
O4—C16	1.3495 (19)	C8—H8B	0.9900
O4—C14	1.4660 (19)	C9—C10	1.512 (2)
O5—C16	1.203 (2)	C9—H9A	0.9900
O6—C19	1.206 (2)	C9—H9B	0.9900
O7—C19	1.337 (2)	C10—C14	1.505 (2)
O7—H7	0.88 (2)	C11—C13	1.321 (2)
C1—C10	1.334 (2)	C11—C12	1.486 (2)
C1—C2	1.507 (2)	C13—H13A	0.9500
C1—H1A	0.9500	C13—H13B	0.9500
C2—C3	1.542 (2)	C14—H14A	0.9900
C2—H2A	0.9900	C14—H14B	0.9900
C2—H2B	0.9900	C15—H15A	0.9800
C3—C4	1.499 (2)	C15—H15B	0.9800
C3—H3A	0.9900	C15—H15C	0.9800
C3—H3B	0.9900	C16—C17	1.503 (2)
C4—C5	1.468 (2)	C17—C18	1.522 (2)
C4—C15	1.504 (2)	C17—H17A	0.9900
C5—C6	1.501 (2)	C17—H17B	0.9900
C5—H5A	1.0000	C18—C19	1.502 (2)
C6—C7	1.541 (2)	C18—H18A	0.9900
C6—H6A	1.0000	C18—H18B	0.9900
			
C5—O1—C4	60.30 (9)	C10—C9—C8	115.61 (13)
C12—O2—C6	110.19 (12)	C10—C9—H9A	108.4
C16—O4—C14	116.01 (12)	C8—C9—H9A	108.4
C19—O7—H7	109.5 (16)	C10—C9—H9B	108.4
C10—C1—C2	128.74 (16)	C8—C9—H9B	108.4
C10—C1—H1A	115.6	H9A—C9—H9B	107.4
C2—C1—H1A	115.6	C1—C10—C14	118.03 (15)
C1—C2—C3	113.86 (14)	C1—C10—C9	124.46 (15)
C1—C2—H2A	108.8	C14—C10—C9	117.48 (14)
C3—C2—H2A	108.8	C13—C11—C12	121.97 (16)
C1—C2—H2B	108.8	C13—C11—C7	130.49 (17)
C3—C2—H2B	108.8	C12—C11—C7	107.54 (13)
H2A—C2—H2B	107.7	O3—C12—O2	121.52 (16)
C4—C3—C2	110.68 (13)	O3—C12—C11	129.52 (16)
C4—C3—H3A	109.5	O2—C12—C11	108.92 (13)
C2—C3—H3A	109.5	C11—C13—H13A	120.0
C4—C3—H3B	109.5	C11—C13—H13B	120.0
C2—C3—H3B	109.5	H13A—C13—H13B	120.0
H3A—C3—H3B	108.1	O4—C14—C10	107.42 (12)
C5—C4—O1	59.36 (9)	O4—C14—H14A	110.2
C5—C4—C3	117.22 (14)	C10—C14—H14A	110.2
O1—C4—C3	115.97 (13)	O4—C14—H14B	110.2
C5—C4—C15	122.24 (14)	C10—C14—H14B	110.2
O1—C4—C15	112.27 (13)	H14A—C14—H14B	108.5
C3—C4—C15	116.31 (14)	C4—C15—H15A	109.5
O1—C5—C4	60.34 (9)	C4—C15—H15B	109.5
O1—C5—C6	118.69 (12)	H15A—C15—H15B	109.5
C4—C5—C6	122.12 (13)	C4—C15—H15C	109.5
O1—C5—H5A	114.9	H15A—C15—H15C	109.5
C4—C5—H5A	114.9	H15B—C15—H15C	109.5
C6—C5—H5A	114.9	O5—C16—O4	123.66 (15)
O2—C6—C5	108.69 (12)	O5—C16—C17	125.95 (15)
O2—C6—C7	105.78 (12)	O4—C16—C17	110.36 (14)
C5—C6—C7	111.72 (12)	C16—C17—C18	114.48 (15)
O2—C6—H6A	110.2	C16—C17—H17A	108.6
C5—C6—H6A	110.2	C18—C17—H17A	108.6
C7—C6—H6A	110.2	C16—C17—H17B	108.6
C11—C7—C8	115.82 (13)	C18—C17—H17B	108.6
C11—C7—C6	101.03 (12)	H17A—C17—H17B	107.6
C8—C7—C6	116.32 (13)	C19—C18—C17	114.15 (15)
C11—C7—H7A	107.7	C19—C18—H18A	108.7
C8—C7—H7A	107.7	C17—C18—H18A	108.7
C6—C7—H7A	107.7	C19—C18—H18B	108.7
C7—C8—C9	116.28 (13)	C17—C18—H18B	108.7
C7—C8—H8A	108.2	H18A—C18—H18B	107.6
C9—C8—H8A	108.2	O6—C19—O7	123.18 (15)
C7—C8—H8B	108.2	O6—C19—C18	125.75 (15)
C9—C8—H8B	108.2	O7—C19—C18	111.05 (14)
H8A—C8—H8B	107.4		
			
C10—C1—C2—C3	−100.0 (2)	C7—C8—C9—C10	−47.66 (19)
C1—C2—C3—C4	75.03 (19)	C2—C1—C10—C14	−175.00 (15)
C5—O1—C4—C3	107.62 (16)	C2—C1—C10—C9	3.1 (3)
C5—O1—C4—C15	−115.31 (15)	C8—C9—C10—C1	128.93 (16)
C2—C3—C4—C5	−85.28 (17)	C8—C9—C10—C14	−53.01 (19)
C2—C3—C4—O1	−152.51 (14)	C8—C7—C11—C13	−34.8 (2)
C2—C3—C4—C15	72.17 (18)	C6—C7—C11—C13	−161.43 (18)
C4—O1—C5—C6	112.70 (16)	C8—C7—C11—C12	146.07 (14)
C3—C4—C5—O1	−105.53 (14)	C6—C7—C11—C12	19.48 (15)
C15—C4—C5—O1	98.46 (16)	C6—O2—C12—O3	171.71 (15)
O1—C4—C5—C6	−107.15 (15)	C6—O2—C12—C11	−10.25 (17)
C3—C4—C5—C6	147.32 (14)	C13—C11—C12—O3	−8.2 (3)
C15—C4—C5—C6	−8.7 (2)	C7—C11—C12—O3	170.97 (17)
C12—O2—C6—C5	143.08 (13)	C13—C11—C12—O2	173.94 (16)
C12—O2—C6—C7	22.97 (16)	C7—C11—C12—O2	−6.87 (17)
O1—C5—C6—O2	57.76 (17)	C16—O4—C14—C10	−175.73 (13)
C4—C5—C6—O2	128.95 (14)	C1—C10—C14—O4	99.00 (17)
O1—C5—C6—C7	174.11 (12)	C9—C10—C14—O4	−79.19 (17)
C4—C5—C6—C7	−114.70 (16)	C14—O4—C16—O5	−3.4 (2)
O2—C6—C7—C11	−25.13 (15)	C14—O4—C16—C17	174.58 (13)
C5—C6—C7—C11	−143.23 (13)	O5—C16—C17—C18	−0.4 (2)
O2—C6—C7—C8	−151.39 (13)	O4—C16—C17—C18	−178.27 (13)
C5—C6—C7—C8	90.51 (16)	C16—C17—C18—C19	−89.65 (18)
C11—C7—C8—C9	176.24 (14)	C17—C18—C19—O6	0.4 (3)
C6—C7—C8—C9	−65.30 (19)	C17—C18—C19—O7	−178.44 (14)

### Hydrogen-bond geometry (Å, º)

**Table:**

*D*—H···*A*	*D*—H	H···*A*	*D*···*A*	*D*—H···*A*
O7—H7···O1^i^	0.88 (2)	1.91 (3)	2.7616 (16)	162 (2)
C6—H6*A*···O6^ii^	1.00	2.60	3.173 (2)	117
C7—H7*A*···O1^iii^	1.00	2.40	3.3293 (19)	154
C14—H14*A*···O7^iv^	0.99	2.51	3.418 (2)	153

Symmetry codes: (i) −*x*+3/2, −*y*+1, *z*+1/2; (ii) −*x*+1, *y*−1/2, −*z*+3/2; (iii) *x*+1/2, −*y*+1/2, −*z*+1; (iv) −*x*+2, *y*−1/2, −*z*+3/2.
